# Spirit of the British and American Periodicals

**Published:** 1842-01-01

**Authors:** 


### Spirit of the British and American Periodicals, &c.

### Delirium connected with certain States of the Heart in Fever. By Dr. Hudson, of Navan.

Dr. Hudson, the author of a valuable dissertation on fever in this Journal, has lately published some observations in our Dublin contemporary on the subject at the head of this paper. In 1840 a putrid or spotted fever prevailed in Navan and neighbourhood, and was very fatal. Generally the symptoms of debility were so prominent that wine and opium were necessary; but in several instances the low muttering delirium changed into that of a furious character, and ultimately into coma, on the exhibition of a small dose of opium. This led our author to make some inquiries into the conditions under which these phenomena occurred. An observation of Dr. Stokes led the way in this inquiry. "*In the diminished impulse, and in the feebleness or extinction of the first sound, we have a new, direct, and important indication for the use of wine in typhus fever.*" Dr. H. says that every day's experience increases his confidence in the value of this mode of diagnosis—and he thinks that wine should never be given in the absence of the above symptom. But the rule for the exhibition of opium has not been laid down so accurately by authors.

"Numerous observations have led me to conclude that opium agrees with that state of the cerebral circulation with which wine agrees, and *vice versâ*, and that the indications derived from the signs of the heart are the same, and of equal value with reference to both. I have already stated that in several instances bad effects seemed to follow the exhibition of opium and tartar emetic. A little observation showed that the same conditions were present which caused wine to disagree, and conversely, that those in which opium produced the best effects were precisely the same as those in which wine, freely administered, did good. In one of these cases the patient took, before sleep could be procured, a drachm and a half of the acetum opii, with six grains of tartar-emetic, in divided doses, and with the best effect, while in another, a single dose of six drops of the same preparation, combined, also, with the antimony, was followed by loss of speech and power of deglutition, tetanic rigidity of the muscles, coma, and death in rapid succession. Here were two opposite conditions of the system in the same disease. The states of the cerebral circulation, in particular, must have been different; but by what external character were these to be recognized? Those which appeared upon the external examination of the patients, in the several instances of each class, were—in the first, the signs of a feeble heart, viz., the loss of impulse and diminution or absence of its first sound; in the second, strong impulse, and distinct and loud sounds.

A corresponding difference of the appearances on dissection was found. In those who during life had manifested the signs of a feeble heart, this organ was softened, and the morbid appearances of the brain were those of venous congestion; in the others, the heart was firm and contracted, and the arteries of the brain were injected. A little consideration will show that the conclusion to which these observations would lead as to the connexion between the opposite state of the heart and corresponding states of the cerebral circulation, is consistent with the pathology of the latter affections, and might, to a certain extent, be anticipated by reasoning; for, admitting the truth of the proposition that 'the pathology of the brain is, in many instances, intimately connected with, and dependent upon the pathology of the heart,'* we would (reasoning *a priori*) infer that cerebral excitement, attended with increased strength and activity of the central organ of the circulation, should be found to depend upon a sthenic or arterial congestion, while a feeble state of the propelling power would lead to more or less stagnation in the venous current, and congestion in those vessels with, what always co-exists with such congestions, *a diminished supply of arterial blood*—this last condition being, in all probability, the true cause of the physiological effects of venous congestion of the brain.

We would thus suppose the existence of two opposite pathological states of the brain, requiring opposite treatment, and yet possessing external characters and symptoms having so close a resemblance as to require frequently more than ordinary powers of discrimination to distinguish between them.

Such a supposition is consistent with the analogy of other affections, especially of 'delirium tremens.' Of this disease every practitioner recognizes at least two varieties, one of which is controlled by opium, with the precision and certainty of a specific; while another case, differing so little in its external characters as to be frequently confounded with it, is exasperated and rendered fatal by this medicine. One requires stimulants, the other bleeding and purging. Dissection reveals passive venous congestion in one case, and determination of blood to the brain or membranes in the other."

Dr. Hudson adduces several cases in illustration, to which we must refer our readers. The stethoscopic indications abovementioned are deserving of especial attention by the practitioner.

---

### MEMOIR OF A GENTLEMAN BORN BLIND AND SUCCESSFULLY OPERATED ON IN THE 18TH YEAR OF HIS AGE. By Dr. FRANZ.

At the birth of this young gentleman (the son of a physician) the eyes were found to present a two-fold defect of organization. Both eyes were turned inwards to a great extent—and cataract existed in both. Towards the end of the second year, keratonyxis was performed on the *right* eye, which was followed by iritis, and wasting of the eye-ball. Within the next four years, two similar operations were performed on the *left* eye, without any success, but with no destruction of the eye. The colour of the opacity, at length, became of a clearer white, and some faint perception of a strong light was experienced by the boy.

Into the long and minute description of the state of this gentleman's eyes in his 18th year (1840) we cannot go. It appeared that the *right* eye was completely amaurotic, and the *left*, which had become atrophied, was the only one considered fit for an operation. The following were the steps taken.

" On the 10th of July 1840, in the presence of Dr. Swaine, and with the kind assistance of Messrs. F. Fowke and F. Steinhaeuser, I made an incision in the cornea upwards, and introducing a pair of fine curved forceps, armed with teeth, into the posterior chamber, I seized the anterior wall of the capsule by passing one of the blades of the forceps into its small aperture, and attempted by pulling it slowly to separate it from its adhesion with the uvea and its peripheral connexion, in which I succeeded without producing a prolapsus of the vitreous body, or tearing the capsule, which I now removed. After this proceeding, a large piece of the lens of an opake colour, probably the nucleus, presented itself in the pupil, which was easily removed from the eye by means of Daviel's spoon; the pupillary aperture then appeared perfectly clear and black. The

---

* Dr. Law on Disease of the Brain dependent on Disease of the Heart, Dublin Medical Journal, No. 50.

patient was now turned with his back to the light, for the purpose of trying a few experiments as to his sight, but from these I was obliged to desist on account of the pain which the light produced in the organ. Both eyes were then closed with narrow strips of court-plaster, and the patient carried to bed. Venesection, local bleeding, fomentations with iced water, continued without intermission for about forty-eight hours, together with the scrupulous observance of the most severe regimen, barely succeeded in keeping down the inflammation, the effects of which in this case, where but one eye offered hope, were much to be dreaded, if it should surpass that degree which was necessary for the healing of the wound in the cornea. This process went on and terminated so favourably, that the cicatrix, situated close to the sclerotica, is now scarcely visible. The patient suffered from muscæ volitantes and from a considerable intolerance of light, pain being produced by even a mild degree of light falling on the closed lids. The muscæ volitantes were greatly mitigated, and the intolerance of light ceased, after the lapse of a few weeks, by the use of proper pharmaceutical remedies, by local bleeding, change of air, &c., and the employment of the ophthalmic fountain of Professor Jungken, which I have fully described in the Medical Gazette, vol. xxvii. p. 444. To promote the development of the power of vision, the use of the fountain was continued twice daily, with Pyrmont-water and latterly with simple spring-water, for the space of three months, when it was discontinued, as it began to irritate the eye."

On opening the eye on the third day, he perceived a blaze of light, and all objects confused and in motion. He could not distinguish any object. The pain forced him quickly to close the eye. Gradual exposure of the eye to light habituated the organ to its stimulus; and when vision became tolerably distinct, all objects appeared so near to him that he was afraid of coming in contact with them, so that he was constantly correcting the sense of sight by that of touch.

On the 21st September, 1840, Dr. F. operated on both eyes for the congenital strabismus. This operation was so successful, that the gentleman's personal appearance was much improved. In November he was able to read the names over the shop-windows, and to tell the time, to a minute, by St. Paul's clock. The tide of human existence, however, in the streets, so confused and confounded him, that at last he could see nothing. By the Spring of 1841, the sight was much improved—and improving. The case, altogether, is very creditable to Dr. Franz, as well as interesting to the profession and the public. The paper is published in the Philosophical Transactions for 1841, Part I.

---

### A new Process for Purifying the Waters supplied to the Metropolis, &c. By Thomas Clark, Professor of Chemistry in Aberdeen.

This is a patent process, the secret of which "will be set forth in the specification." So was the secret of Dr. James, but nobody could ever make the fever powder by that specification. We learn, however, that the Thames water is to be purified by means of quick-lime. The state of purity to be attained by the new process, is that which would be effected by "*heating the water till it boiled, and kept at the boiling point for two hours.*" Thus then the muddy and turbid *infusion* of dead dogs, cats, rats, garbage of hospitals and shambles, washings of water-closets, drainings from common sewers, and all utterable and unutterable abominations, is to be brought to the state of a homogeneous *decoction*, by two hours' boiling, and this hell-broth is to be offered to the inhabitants of the metropolis as a PURIFIED WATER!!

The great object and anxiety of the patentee seem to be the precipitation of the chalk from the Thames and River water. Thus, an experiment is stated, in which it is reckoned that two pounds of chalk are partly dissolved and partly suspended in 540 gallons of metropolitan water, and that when the patent process is applied, "*both pounds of chalk will be found at the bottom after subsidence. The* 540 *gallons of water will remain above, clear and colourless, without holding in solution any sensible quantity either of caustic lime or bicarbonate of lime.*"

So, then, we are to swill and swallow *contentedly* the essences of every kind and species of filth that the most fertile imagination can conceive, in gratitude for having got rid of a few grains, daily, of chalk!!!

We shall here take leave of our ingenious patentee, by making him a proposal, which, we sincerely hope, he will accept. Let him leave us the CHALK in the Thames water, and take all the other ingredients—FILTH, SOIL, and SORDES, to himself—as remuneration for the trouble of his chemical purification.

---

### PHYSIOLOGY OF THE VOICE.

"Exceptio probat regulam."

Mons. Reynard has published a case in the Gazette Medicale, where a galley-slave attempted suicide by cutting his throat by transfixing the larynx, from side to side. There was considerable hæmorrhage. The œsophagus was wounded, but not severed. Sustenance was conveyed into the stomach by means of a tube. The subsequent inflammation closed up the air-passage above the wound. He breathed entirely by the laryngeal opening, when a tube was established. M. Reynard made experiments to ascertain whether any air could be expelled or inhaled through the glottis, and concluded that the natural passage was completely closed. Yet speech remained tolerably distinct.

We doubt the fact, and suspect a fallacy. Mr. Price, a jeweller at Portsmouth, was operated on by the late Dr. Denmark, and Dr. Johnson, in a case of laryngitis, when the patient was in articulo-mortis. The trachea was opened, and Mr. Price breathed through the tube from that day to this (for we believe he is still alive) some 25 or 26 years. Now here, although the passage was not entirely closed, for a very small stream of air could be forced through the glottis, yet the power of articulation was lost. With this fact before our eyes, we cannot assent to the correctness of M. Reynard's statement. Were we to believe that a man could speak without the organ of voice, or hear without the organ of audition, we ought, in common justice, to believe that Miss Okey saw through her navel, and that the doctor's "bottle-imp" could foretell the issue of a malady, without seeing the patient, or having the slightest acquaintance with the anatomy, physiology, or pathology of diseases!

---

### PARTIAL HYPERTROPHY OF THE HEART. By DR. JOHNSON.

By the term which I have used, I mean a diminished size of the heart, as a whole, while certain portions of it are in a state of hypertrophy. It corresponds with the concentric hypertrophy of authors.

The Hon. Mr. R——, about 40 years of age, came under my observation some five or six weeks before his death. He was greatly emaciated—sallow in his complexion—and with extremely feeble circulation. The pulse was like a thread, at the wrist, and the action of the heart was scarcely audible. The appetite was nearly wanting, and as food produced distressing feelings, he had long adopted a very rigid and abstemious diet, which probably tended to increase the emaciation. I examined him with the greatest care from head to foot, without being able to detect any lesion that could account for the progressive waste of flesh.

The patient (if he could be called such) went direct to Leamington, and never took any thing which I prescribed. He placed himself under Dr. Jephson, who gave favourable hopes to his family of ultimate recovery. A month's treatment, however, produced no amendment, and Mr. R. returned, with great difficulty, and in the most exhausted state to London, when he came again under my care. The emaciation was now at its utmost extreme, and as he could take very little nourishment by the mouth, he was supported by strong beef-tea thrown up into the colon every eight hours. The pulse was now scarcely perceptible at the wrist, and the action of the heart inaudible in any part of the chest. He had no cough—clean tongue—intellects unclouded—and, after lingering about a week, he ceased to exist—pale and pulseless, like a person expiring of cholera.

On dissection (by my son) 24 hours after death, all the organs and structures in the body, with one exception, were sound. On opening the chest, the lungs, on both sides, completely collapsed, leaving in view a heart of the most extraordinarily small dimensions. The pericardium was intimately glued to the heart, so as to be incapable of separation, so ancient and complete had been the adhesion. The heart itself was not more than half its natural size; yet its parietes (ventricular) were full an inch in thickness. This diminution of general size, with hypertrophy of the walls, had so encroached upon the cavities, that the left chamber could not contain more than three tea-spoonfuls of blood! The consequence was, that at each ventricular contraction about one-fourth of the normal or natural quantity of blood was discharged from the heart into both aortic and pulmonary system. The general emaciation of the whole body thus so very imperfectly supplied with the vital fluid, is readily accounted for. In respect to diagnosis during life, I conceive that in a case of this kind, and to this extent, it would require more diagnostic means than we yet possess to detect the true nature of the organic lesion.

---

### CARLISLE.

### Periodical *versus* Permanent Duty in the Medical Charities of Carlisle.

A sharp discussion and correspondence have lately taken place between the medical gentlemen of Carlisle and the Governors of the Dispensary and of the Fever Hospital of that place.

The great majority of the medical practitioners of that city proffered their *gratuitous* services to the above institutions, coupled with the proposal that *all* the said practitioners, who volunteered their services, should have periods of duty, *in rotation*, assigned them, instead of that duty being *permanently* allotted to certain individuals elected for that purpose. To this proposal the Governors demurred: but, whatever reasons or arguments they may have employed among themselves, there is nothing like either reason or argument broached in the official correspondence.

The principle of rotation might not, indeed, prove applicable to, or practicable in, the metropolis, or in very large provincial cities, where the number of medical practitioners happen to be out of all proportion to the number or extent of the charitable institutions: but in such a place as Carlisle, containing, perhaps, not more than 20,000 inhabitants, the plan is perfectly feasible, and would, we conceive, prove eminently beneficial to the sick, to the public, and to the profession. There could not be the slightest reason to apprehend that unqualified individuals would volunteer their services in a public institution, where exposure would as certainly follow error or ignorance, as the shadow follows the substance. By this plan, knowledge would be more equally diffused among the medical practitioners of the place, and surely this diffusion would extend its beneficial influence to the lay inhabitants themselves, when on the bed of sickness. The only plausible objection to this *rotation duty* might lie in the idea that the same patient might, in the course of treatment, come under two or more medical practitioners. This objection is perfectly futile. It is every day refuted by patients in private life, who naturally and properly change their medical attendants when they think they are not deriving so much benefit as they expected from remedial agency. And why should the poor be deprived of an advantage, real or imaginary, which the rich prize so dearly? The fact is, that such changes in public hospitals and infirmaries would very often be productive of the best effects to the suffering inmates. We can only make room here for the following conclusion of an admirable address from the Medical Practitioners of Carlisle to their High Mightinesses, the Governors of the Infirmary.

" By accepting, then, the services of all the resident Medical practitioners who may be willing to devote a portion of their time and labour to the duties of the Hospital, the Governors would give to each a permanent and an active interest in its prosperity. By thus creating among them, at the same time, an earnest spirit of emulation, and a desire on the part of every man to discharge his duty in the manner most conducive to his own credit, they would certainly obtain the best pledge for his watchful attention to the interests of the sick. The perfect freedom of intercourse among the Medical Officers of the Hospital would necessarily foster a more extended desire for scientific enquiry and observation. Their common stock of practical information would be consequently increased; and under circumstances where no unseemly or disparaging jealousies would be likely to interrupt the general co-operation in a design of so much importance, a higher tone of professional feeling would as certainly be produced. Situated as the Infirmary is, at no inconsiderable distance from the town, the continual necessity for daily attendance during every season of the year, would unquestionably become irksome to the medical men, were the appointments limited to a few; but this inconvenience would be completely obviated by the division of a larger number into successive sections. The recurrence of the periods of active service would be looked forward to by each with an interest perpetually fresh. Every remarkable circumstance would be open to the observation of all; and in cases of peculiar doubt, danger, or emergency, the resource of a general consultation would at all times be promptly available.

Even were there no *superior* advantages presented by the arrangements which we propose, we conceive that a wish on the part of those who in either case would be appointed, to be associated with their professional neighbours, and to share with them in the labours and the advantages of the institution, ought to be regarded by the Governors as a proof of the earnest sincerity with which they would endeavour to carry out its design, and an ample guarantee against those selfish and sordid motives which have been too often known to interfere with the fulfilment of a similar purpose.

(Signed)

WILLIAM JACKSON, M.D.
THOMAS ELLIOT, Surgeon.
RICHARD JAMES, M.D.

JAMES MARRS, Surgeon.
R. ATKINSON, M.D.
EDWARD BOWMAN, Surgeon.

R. Oliver, Licentiate Royal College of Physicians, London.

Peter Linton, Surgeon.

William Elliot, M.D.

Francis W. Kerr, Surgeon.

John Mortimer, Surgeon.

Joseph Cartmell, M.D.

John Hodgson, Surgeon.

William Dalton, M.D.

Wm. Nicholson, Surgeon.

Wm. Jackson, Chairman.
R. James, Secretary."

It is hardly necessary to state that the voluntary and gratuitous services of the above highly respectable practitioners were declined, and we believe a *stipendiary* surgeon has been engaged to perform duties in an institution supported by subscriptions, and which might have enlisted in its favour the whole influence and abilities of the medical corps of the city, without expense!

---

### Institution for the Treatment of Curvatures of the Spine, &c.

Few in the Profession, or out of it, are aware of the existence, in this country, of an Institution for the Treatment of Spinal Diseases, so perfect as to rival the best establishments in France. Such an Institution there is in the immediate neighbourhood of London, and it comprises every requisite, mechanical and general, for the efficient management of those complaints. The place to which we allude is Vanburgh Castle, contiguous to Blackheath. This spacious mansion built by Sir John Vanburgh, has been taken by Dr. Potts, and within its walls, as well as on its lawns and pleasure grounds, contrivances of the most ingenious and appropriate description have been laid out for the use of spinal patients. Planes for reclining, reading, working, playing at the piano—apparatuses for exercising the various muscles—in the recumbent or any other posture—swings and all kinds of calisthenic instruments—baths of every sort—may be found there. Besides, the corporeal, there is mental discipline, the establishment being educational as well as remedial. Mrs. Potts and her daughters superintend the former, assisted by the best masters in all the branches of knowledge and accomplishments cultivated by females. A lady from Dresden, who teaches the German language, is resident in the house.

It is impossible to do justice to the scientific acuteness and mechanical talent displayed by Dr. Potts in his arrangements. Every apparatus is made on the premises, where there is a turning lathe, a forge, &c. superintended by himself. He is a first-rate mechanic, and not only directs but can execute.

We know of no pleasanter excursion for a medical man than that to Vanburgh Castle. The admirable character of the arrangements, the absence of charlatanery, which so often pervades these kinds of establishments, the beauty of the spot, and the frank hospitality of the host, are attractions that it is impossible not to feel.

The Institution deserves the warmest encouragement on the part of the profession, and the liberal patronage of the public; never before were there such means in this country for remedying spinal disorders. To be known is all that is necessary for its reputation and success.

---

### Phrenology.

*(To the Editor of the Medico-Chirurgical Review.)*

Newtownards, November, 1841.

Sir,—If you think the following remarks, confirmatory of some of the doctrines of phrenology, worthy of publication, I will feel honoured by your giving them a place in your valuable Journal.

I am, your obedient servant,
D. JAMISON, M.D.

### DEFICIENCY IN SIZE AND DISEASE OF THE CEREBELLUM, THE CAUSES OF ANAPHRODISIA.

Since I became acquainted with the science of phrenology, some years ago, my attention has been directed to the condition of the cerebellum in those individuals who have consulted me for impotence, and in others. I have attentively observed and studied a great number of cases, and am led to regard the following conclusions as correct. I hope their publication may have the effect of directing medical practitioners more closely to observe the undoubted connexion which exists between the state of the genital organs and the cerebellum, and a disease which in its various degrees is much more prevalent than is commonly imagined, and is the cause of a great many evils.

1. The sexual passion has its seat in the cerebellum, and is energetic or the reverse in proportion to the size and tone of this organ.

2. Smallness of the cerebellum, much inequality of its lobes, and deficiency of its tone, are the causes of impotence.

3. When the cerebellum is very small, impotence is permanent.

4. When the cerebellum is small, it soon suffers in tone if made to perform its functions with ordinary frequency.

5. When one lobe of the cerebellum is small and the other large in a man, it is sometimes the case, that he, at intervals distant in proportion to the size of the large lobe, performs the generative act imperfectly, until the large lobe, which had been exhausted, recovers its tone.

6. When the cerebellum is very large, and is much exerted, as it usually is in such cases, it becomes impaired in tone, and impotence is sometimes the result; but the generative act may be well performed by a large cerebellum, even when impaired in tone.

7. Average endowment of the cerebellum is most favourable to permanent potency.

8. When the cerebellum becomes much deficient in tone, if it be not soon cured, the spinal marrow and its nerves, the organic nervous system, the intellect, and moral feelings are successively debilitated.

9. Deficiency of tone of the cerebellum in the male or female is often transmitted to the offspring.

10. Impaired tone of the cerebellum is the cause of spermatorrhea.

11. The size of the genital organs exercises no influence on their activity or vigour, they are often inert when large, and vigorous when small.

12. The father of a monstrosity, an account of the post-mortem examination of which I published sometime ago, had the cerebellum small and debilitated, and had also spermatorrhea, he was permanently weak in the genital organs, and was the means of making me acquainted with many similar cases, and their peculiar symptoms. His wife became jealous and went mad in consequence of believing that he was unfaithful, and that what was the result of deblity, was caused by dislike of her. She died in a lunatic asylum. These facts, in connexion with remark No. 8, render it probable, in my opinion, that the subjects of abnormal organization are the products of parents whose generative apparatus was diseased; and general health consequently much impaired. I think the condition of the cerebellum in the parents of monstrosities should be observed.

13. Permanent or frequent impotence, or even continued partial debility of the genital organs, in men who have large self-esteem and destructiveness, and benevolence and conscientiousness not very large, often produces strongly selfishness and malignity; and also cunning and falsity; for though secretiveness should not be large, it is so much exercised in these cases to conceal the symptoms of their disease and preserve the reputation of virility, that it operates as if it predominated in size. This is in accordance with the remark of Dr. Cox, "that it seems to be a law of the human constitution that when any of the faculties is pained or disagreeably active" destructiveness instantly comes into play. Here amativeness is mortified, and self-esteem and love of approbation disagreeably active, and destructiveness becomes consequently excited, secretiveness being active also, malevolence, cunning, and falsehood result.

14. Over exertion or exhaustion of the cerebellum robs adhesiveness and combativeness of their power and thus causes cowardice.

15. Whatever exhausts the power of the constitution, seems not only to diminish the power of combativeness, but also to stimulate cautiousness.

16. In some men an activity and power of the cerebellum greater than what we would expect from their temperaments and developments may exist for a long time without producing impotence; here it seems to appropriate more than its own share of the nervous energy of the system; the other organs of the body suffering a diminution of power apparently that the generative apparatus may obtain an increase.

17. The cerebellum is in general too much exercised in the married state.

18. When the cerebellum is too much exercised, no matter what the size of it may be, it becomes impaired in tone.

19. Men and women who have the cerebellum much below the average size should not marry.

20. Impotence is curable in all cases but where the cerebellum is very small or disorganized.

21. Fluor albus is caused by deficient tone of the cerebellum in many cases.

22. Deficient size and tone of the cerebellum in males or females is a cause of want of liveliness, and sometimes of melancholy and madness.

23. Disease of the cerebellum is often the real cause of absurd eccentricities.

24. The treatment of impotence should always be directed with a view to its origin in the cerebellum.

---

### Nitrate of Silver in Gonorrhœa.

Mr. Carmichael, of Dublin, has strenuously repudiated the practice attributed to him by Mr. Acton, of using injections of nitrate of silver, so strong as ten or twelve grains to the ounce of water. In Mr. C's published work on the venereal disease, he stigmatizes the above mode of treatment, as "*a practice that cannot be too strongly deprecated.*" In a more recent publication—a lecture—he states that he has used the injection, but never in a greater degree of strength than one grain to the ounce—more frequently a quarter or half a grain to that quantity.

---

### Suicide and Cross-way Burials.

It appears that a little while ago there was a sort of suicidal epidemic in the city. Night after night persons threw or tried to throw themselves into the Thames. Sir Peter Laurie determined to punish those who failed in their attempts at drowning. They were imprisoned, sent to take their trial and so forth, and the epidemic subsided.

On this text our contemporary, the Medical Gazette, enlarges, and though we think there is much good sense, we also think there is much that is questionable in his remarks. The gist of them is contained in the following passage.

"Need we point out how these cases afford another proof, though of a very different kind from those commonly adduced, of the propriety of treating those who are, or who seem to be, insane, in the same manner as if they were thoroughly rational? It is quite plain that for all these cases, the best method of preventing suicide is, to treat it as a punishable offence, that is, as the evil deed of a responsible man; to make no difference, *primâ facie*, between suicide and homicide. They show, also, the power which is in the hands of coroners' juries to put a check upon this fearful practice by bringing in, in every case where the contrary is not clearly indicated, such a verdict as may lead to the body of the responsible self-destroyer being deprived of that respectful treatment which seems to all men an object of anxious desire. No sympathy can be more unsound than that which dictates men to believe a suicide *ipso facto* insane; nor can any be attended with worse effects. Make all suicides objects of pity, of sympathy, and of respect, and they will not be rare: but put them on the level with homicides, let the bodies of all be disgraced in whose cases there are not plainly circumstances that proved them to be irresponsible to themselves (and, for their last deed to their Maker), and then will the number of these criminals diminish yearly. In a word, let juries act towards the dead in the same spirit as the magistrates have acted to the living, and they will soon very materially diminish their own and the coroners' labours."

We think that the danger of acting literally on our contemporary's advice in the treatment of mania must be obvious. A man who is confessedly deprived of reason is to be amenable to punishment; like him whose reason is intact. It would be equally difficult to shew the justice and humanity of this proposal. Punishment implies crime—crime the power on the part of the criminal to distinguish between right and wrong—and the loss of reason abrogates that power. A great proportion of our punishable offences are not stamped as such by any natural laws, but defined by highly cultivated reason, or by the revealed will of the Almighty. To punish the irrational for sinning against what it required reason to create, and implies reason to observe, is harsh and unjust indeed. We grant the occasional difficulty that exists, in determining the requisite quantum of insanity, but that is a question of detail, and excluded from the broad principle laid down by our contemporary.

No doubt there are certain cases, like the fashion for drowning in the City, where law may thrust its arm between the act and the actor. But these are samples of a pseudo sort of mania, and admit of a special treatment.

Nor can we quite agree with our contemporary in his advice to coroners' juries. The stake and the cross-way burial would be revived were his suggestions to be acted on. But we would remind him that this is no new method—*it has been tried and failed.* The intelligence of the age put it down. It proved, we apprehend, in practice unavailing in deterring from the guilt, powerful only in injuring the innocent. The feelings of survivors are lacerated sufficiently by the dreadful act itself, without any posthumous addition. Are the unhappy widow and the helpless children of the suicide to be tortured by the reflection that as the husband and the father's death was dreadful, his corpse is spurned as infamous? Experience has proved the inutility, our feelings proclaim the injustice of this. Would all the horrors of the old suicidal code of England have prevented Romilly from committing self-destruction, or would enlightened Europe have tolerated their infliction upon his remains? We fancy not. Then if insanity is to save from the coroner's vengeance the bodies of the rich, are only the poor to be consigned to it? Is Castlereagh to repose beneath his gorgeous pall, and some wretched victim of seduction to be staked? These are some of the difficulties that they would have to grapple with, who should venture to resuscitate the penal treatment of suicide.

### Surgeons entitled to Payment for Medical Attendance.

The following case, recently decided in the Court of Common Pleas, under the direction of Chief Justice Tindal, is important.

Mr. Baxter, a surgeon of some eminence, brought an action against Gray and another, as executors of a Mrs. Bostock, to recover the sum of £569. 2s. for a long attendance on that lady, who died at the age of 90. The plaintiff, Mr. Baxter, practised as a surgeon: he did not, however, compound or send in medicines, but merely prescribed as a physician. He had attended Mrs. Bostock constantly from the year 1829 to 1835. It appeared from the evidence that Mr. Baxter calculated on a *post-mortem* remuneration in the shape of annuity or legacy, and did not receive an immediate *honorarium* for each visit. Mrs. Bostock died, but the expected legacy was a "castle in the air;" an action was, therefore, brought to recover for attendance. The defence set up was, that the plaintiff had been paid for his attendance, but only £33. were proved to have been paid.

The facts which we have just stated having been established, Lord Chief Justice Tindal told the jury that there were only two questions for their consideration; first, the amount to which the plaintiff was entitled for attendance; and secondly, whether the whole or any part of that amount had been paid. The evidence was very loose on the part of the plaintiff, to show the precise number of attendances which had been given, but there could be no doubt that *the attendance was considerable,* and that the plaintiff was *entitled to something handsome for it.*

The jury returned a verdict for the plaintiff—damages £217.

Our contemporary, the Provincial Medical Journal, observes very justly, that if a surgeon can recover for medical attendance, a physician ought to do so too. This certainly is reason if it should not prove to be law.

---

### Homœopathy Exposed.

The papers have been circulating the following paragraph.

"The Duke of Canizzaro died from taking three pills at once, ordered to be taken singly, either through his own mistake, or through that of his *homœopathic* physician, and that these pills contained *arsenic.* Thus we see a nobleman, in the enjoyment of a large fortune, dying, *poisoned like a rat.* Considering these pills were prescribed in conformity to *homœopathic* practice, in which only *millioneth* doses are supposed to be used, so that a few hundred thousand portions might be taken without producing death, one can but look upon this result as no less extraordinary than unfortunate. It gives rise to no little matter of reflection upon the source of the active effect of these doses of fabulous diminutiveness, and it shows that those optimists may err who think that homœopathy is a mere hocus-pocus, like the *papato* of the seventeenth century."

We have always thought and said that the clever rogues among the homœopathists take good care to give active doses of medicine under cover of their infinitesimal humbug. Here is a case in point. How could the Duke of Canizzaro die from swallowing two or two hundred millioneths of a grain of arsenic? The quackery and imposture of the thing are palpable. But it is of no use telling the public to avoid quacks. They *will* be gulled, and therefore individuals, like the Duke of Canizzaro, must pay for it.

---

### Pharmacy in France.

The School of Pharmacy in Paris comprises five titular professors, "*professeurs*

*titulaires*," and three assistant professors, "*professeurs adjoints*." The other schools have three titular and two assistant professors. In each school there are also associated assistants, "*agrégés*," appointed for five years, who take the place of the professors in case of their absence, and assist at examinations. In the school in Paris there are five associate assistants, and three in the schools of Montpellier and Strasbourg. The titular and assistant professors are appointed by the minister of public instruction, from a double list of presentations, made, the one by the *School of Pharmacy*, and the other by the *Faculty of Medicine* of the town in which the school is situated. Each list of presentations contains the names of two candidates, but the same candidates may be presented both by the School of Pharmacy and by the Faculty of Medicine. No one can be named as titular professor who is not a doctor in physical sciences, and thirty years of age. The assistant professors are required to be licentiates in physical sciences, and twenty-five years of age. Both are required to have been admitted Pharmaciens in one of the schools of Pharmacy. The associated assistants are to be appointed by "*concours*," in a manner to be hereafter arranged in the council of public instruction. To be admitted to the "*concours*," it will be sufficient to produce the diploma of a Pharmacien and of a bachelor in physical sciences. The director of the school is to be chosen by the minister of public instruction, from among the titular professors. He is to be in office for five years, and is eligible for re-election. Each school is provided with a responsible secretary, chosen by the minister of public instruction, from among the titular or assistant professors. There are also one or more "*preparateurs*," who must have the degree of bachelor of physical sciences, and are appointed by the director, with the concurrence of the professors. The director appoints the officers and servants. The *instruction* in each school, comprises :—

*First year.*—Physics, Chemistry, and the Natural History of Medicines.

*Second year.*—Natural History of Medicines, Materia Medica and Pharmacy, properly so called.

*Third year.*—Toxicology; and in the practical school, Chemical and Pharmaceutical manipulations.

No candidate can be admitted to an examination for the title of Pharmacien, who has not obtained the degree of bachelor of letters. Besides the two professors in medicine who are appointed to officiate at the examinations, three members of the College of Pharmacy must also be present, namely, two titular or assistant professors, and one associated assistant. The students of the schools of Pharmacy, who have gained prizes at the "*concours*," are exempted from the fees. The amount remitted for each prize is to be regulated by the university. The names of the successful students are published.

The *receipts and expenditure* of the schools of Pharmacy are carried to the national budget of public instruction. The titular Professor, in Paris, is to receive a fixed annual salary of 4,000 francs; in the departments of 3,000 francs. The Assistant Professors, in Paris, are to receive an annual salary of 2,400 francs, in the departments 1,500 francs. The Director is to receive in addition, as a jointure, an annual stipend of 1,500 francs, in Paris, and 1000 francs in the other Colleges. The salary of the Secretary, in Paris, is 3,000 francs: in the other schools, 1,500 francs. The salary of the Preparateurs, is 1,200 francs. The payment for attendance at the examinations is 10 francs for those functionaries who are called upon to officiate. The same is allowed to the Professors, who are charged with the examination of herbalists. The fee for the annual certificate, granted to each student, is fixed at 36 francs in each of the schools. The charge for examinations remains unaltered; for the first examination, 200 francs; for the second, 200 francs; for the third, 500 francs. The expenses of operations and demonstrations, incurred during the third year, which are defrayed by the candidates, are fixed at 200 francs, in Paris, and 150 francs in the other schools.

The acquirement of the diploma of Bachelor of Letters, will not be required in the candidates for examination, until the 1st of February, 1844.—*Pharmaceut. Transactions, Oct.* 1. 1841.

---

### Pharmacy in England.

In the Pharmaceutical Journal we find the following outline of the plan in contemplation with respect to our future chemists and druggists. The "College of Pharmacy," if it is ever raised, will be due in a great measure to the energy, enthusiasm, and perseverance of Mr. Jacob Bell. We think that his exertions bid fair to elevate the social as well as the scientific position of the trade.

Three examinations are proposed.

1st. Apprentices should be examined in the classics and other elementary knowledge, which constitutes an ordinary liberal education. It would be desirable to inculcate the advantage of including the rudiments of physics and natural philosophy in the academical studies of the future Chemist, which would not only prepare his mind for the reception of the store of information required in his business, but would also be the means of testing his faculties, and ascertaining how far the practice of Pharmacy would be suited to his disposition. The examination of Apprentices will serve to restrict the adoption of the pursuit to those whose abilities and station in life are such as to afford a prospect of credit and success.

2nd. At the expiration of their apprenticeship or pupilage, they should be examined, in order to become Associates in the elements of chemistry and materia medica, botany, and the compounding of prescriptions. But a young man may be competent to perform the duties of a dispensing assistant and to retail drugs, without possessing that extensive practical and theoretical acquaintance with his business, which would be expected in a principal who is responsible for the superintendence and management of a pharmaceutical establishment.

This implies the necessity for a

3rd. Examination for the higher degree, which would entitle him to be admitted a Member of the "College of Pharmacy." This examination would comprise a more extended knowledge of chemistry, botany, materia medica, and practical pharmacy, including operations and demonstrations, and, probably, toxicology.—*Pharmaceutical Transactions, Oct.* 1, 1841.

---

### Proposed Scale of Fees of General Practitioners.

A correspondent of the Provincial Medical and Surgical Journal suggests the following:—

| | *s.* | *d.* |
|---|---|---|
| For every mile we travel out from home, whether in the service of rich or poor - - - - - - - - | 1 | 6 |
| For every attendance upon a pauper - - - - - | 1 | 0 |
| For bleeding a pauper, or dressing a wound, not associated with compound fracture, or other very serious case - - - - | 1 | 0 |
| For attendance in the family of a labourer not a pauper - - | 1 | 6 |
| For ditto in the family of a small tradesman - - - - | 2 | 6 |
| For ditto, ditto, a grade higher - - - - - | 3 | 6 |
| For ditto, ditto, first class, within three miles - - - - | 5 | 0 |
| For ditto, ditto, exceeding three miles, not exceeding five miles - - | 7 | 6 |
| For ditto, ditto, exceeding five miles - - - - | 10 | 0 |
| For extraordinary attendance at the rate of per hour - - - | 7 | 6 |

We fear it will be some time before any thing so just as the above will be obtained. But our brethren should constantly aim at payment *in* fees, and not *for* drugs.

